# Does Ploidy Level Directly Control Cell Size? Counterevidence from Arabidopsis Genetics

**DOI:** 10.1371/journal.pone.0083729

**Published:** 2013-12-12

**Authors:** Hirokazu Tsukaya

**Affiliations:** Department of Biological Science, Graduate School of Science, The University of Tokyo, Bunkyo-ku, Tokyo, Japan; University of Antwerp, Belgium

## Abstract

Ploidy level affects cell size in many organisms, and ploidy-dependent cell enlargement has been used to breed many useful organisms. However, how polyploidy affects cell size remains unknown. Previous studies have explored changes in transcriptome data caused by polyploidy, but have not been successful. The most naïve theory explaining ploidy-dependent cell enlargement is that increases in gene copy number increase the amount of protein, which in turn increases the cell volume. This hypothesis can be evaluated by examining whether any strains, mutants, or transgenics show the same cell size before and after a tetraploidization event. I performed this experiment by tetraploidizing various mutants and transgenics of *Arabidopsis thaliana*, which show a wide range in cell size, and found that the ploidy-dependent increase in cell volume is genetically regulated. This result is not in agreement with the theory described above.

## Introduction

The ploidy level is known to be closely associated with cell volume in fungi, plants, and animals [1,2]. Polyploid individuals often develop larger bodies due to their increased cell size, and this effect has been used to improve and breed economically useful plants [3] and animals [4]. However, this ploidy-dependent increase in size is neither linear nor constant. For example, hexaploids typically have the maximum body size in an autopolyploidy series [5-7], although cell size is known to increase proportionally with the increase in ploidy level. This suggests that high ploidy levels decrease the number of cells per organ. This “high-ploidy syndrome” is also commonly observed in the model plant *Arabidopsis thaliana* (L.) Heynh. (hereafter, Arabidopsis). In this plant, octaploids are much smaller than tetraploids and even diploids in terms of body size [8], indicating that body size is not passively regulated by ploidy level.

 How then is the cell volume regulated by the ploidy level? This question has been long discussed but the answer remains unknown (reviewed in 8). The most naïve hypothesis is that increases in gene copy number increase the amount of protein, which in turn increases the cell volume. However, the cell structure is composed of linear structures (e.g., DNA, RNA, microtubules), dimensional planes (various membrane structures), and three-dimensional structures (e.g., vacuoles, cytosol). To double the cell volume without changing the functional structures, the amount of all protein species cannot be doubled as discussed in earlier studies [9]. That is, doubling the volume should be accompanied with a 2^2/3^ (= 1.587)-fold increase in two-dimensional structures such as membranes, and a 2^1/3^ (= 1.260)-fold increase in linear structures such as cytoskeletal structures. If all components were doubled, the amount of linear and two-dimensional components would be excessive. This may explain why yeast tetraploids are sensitive to mutations in the chromosome-segregation machinery [9], where the linear, dimensional, and three-dimensional structures must simultaneously and precisely cooperate. This multidimensional problem may also explain why autopolyploid animals are rare in nature [9,10].

 Polyploidy is very common in plants. Approximately 35% of vascular plant species are believed to have polyploid variants [11]. In addition, artificial polyploids can be made by treating plantlets with the microtubule-dissociating reagent colchicine [12]. This allows us to study the mechanisms of polyploidy-dependent changes in organisms. However, in prior studies, microarray analyses of tetraploid/diploid sets of various Arabidopsis accessions did not detect any common accession-independent changes in transcriptome profiles between the tetraploid and diploid [13]. In contrast, the expression levels of specific genes encoding cell-surface components are known to be regulated in a cell volume-dependent manner [14], indicating a possibility that the body size is not passively regulated by the ploidy level.

 Previously, Breuer et al. [15] reported that a defect in cell expansion and organ development in loss-of-function mutants with a severe defect in endoreduplication can be overcome by tetraploidization, indicating that endoreduplication (genome duplication without mitosis) and polyploidization (genome duplication associated with a duplication in the number of chromosomes) share common effect(s) in the regulation of size and differentiation of cells and organs in Arabidopsis. However, we observed that tetraploidization does not result in a doubling of cell volume in the mutants examined above. If the ploidy-dependent increase in cell size is a direct and passive result of an increase in the gene copy number, any strains, mutants, or transgenics should show the same increase in cell volume after tetraploidization. Here, we explored this hypothesis by tetraploidizing various mutants and transgenics of Arabidopsis.

## Results

### Impact of tetraploidization on the size of somatic cells

In this study, 18 Arabidopsis lines with abnormal cell sizes were examined. First, the paradermal cell area in the subepidermal layer was measured for the first set of mature leaves (Table 1). The projected cell area at the diploid state varied from an average of 406.3 (*bin4-1*) to 4782.3 (*cycA2;3*) µm^2^, covering almost all possible cell size variations among mutants of the arabidopsis Columbia background [16]. In wild-type, the ratio of tetraploid cell area to diploid cell area (the 4C/2C ratio) was 1.76 (Table 1). This value was similar to the predicted value based on an assumption that the tetraploid cell volume is twice that of the diploid cell volume and that cell expansion is not polarized (2^2/3^ = 1.587). If ploidy level is directly associated with cell volume, the 4C/2C ratio must be fixed near this value, irrespective of the genetic background. However, as shown in Table 1, this ratio varied significantly from 1.22 to 2.73.

**Table 1:** Cell size and the 4C/2C ratio of the first set of foliage leaves.

Strain	Cell size (µm^2^)*		4C/2Cratio
	Diploid	Tetraploid	
Columbia wt	3041.4 + 99.72	5359.2 + 349.8	1.76
*brassinosteroid insensitive(bin)4-1*	406.3 + 10.3	1067.7 + 37.5	2.63
*root hairless (rhl)2-1*	506.4 + 18.7	1374.4 + 90.7	2.73
*extra small sisters (xs)5-1*	1440.1 + 67.2	2445.3 + 298.9	1.70
*xs10-1*	1448.9 + 111.9	3329.3 + 230.7	2.30
*angustifolia (an)-1*	2082.4 + 33.2	3054.5 + 145.7	1.47
*asymmetric leaves (as)2-1*	2087.3 + 16.8	3825.7 + 324.5	1.84
*xs3-1*	2516.0 + 173.4	4571.0 + 390.9	1.82
*rotundifolia (rot)3-1*	2544.6 + 271.9	3947.2 + 445.2	1.55
*xs6-1*	2559.5 + 235.9	3667.1 + 465.3	1.43
*rpt2a-1*	2624.7 + 299.3	5286.5 + 284.7	2.01
*xs4-1*	2687.7 + 360.3	4504.3 + 707.4	1.68
*xs7-1*	2753.2 + 153.6	4453.9 + 575.4	1.62
*siamese (sim)-1*	2784.4 + 102.2	4787.2 + 689.3	1.72
*26S proteasome regulatory particle subunit (rpn)12-1*	2842.4 + 562.3	5469.4 + 589.8	1.92
*xs1-1*	2903.0 + 289.9	4595.8 + 835.5	1.57
*an3-4*	3139.3 + 259.6	5525.3 + 300.4	1.76
*fugu2-1/fasciata (fas)1-5*	4617.0 + 631.4	5639.1 + 872.4	1.22
*cyclin (cyc)A2;3*	4782.3 + 75.3	6996.2 + 1058.3	1.50

Strains are ordered from those with the smallest cells to the largest. Data are shown as the means + standard deviation. * At least 21 cells were measured for one leaf and at least three leaves were measured from each strain. Values are shown as averages.

 This ratio did not correlate with the cell size in the diploid state. In this state, some mutants and transgenics are known to exhibit “compensation,” or enhanced cell expansion triggered by defective cell proliferation [8,17] (i.e., *an3-4*, *fugu2-1/fas1-5*; [18,19]). The occurrence/absence of compensation was found to be unrelated to the magnitude of the 4C/2C ratio, which varied from 1.22 to 1.76 among the above compensation-exhibiting strains (Table 1). Since compensated cell enlargement is often independent of the endoreduplication process [20], ploidy-dependent cell expansion may occur in additive to the compensated cell enlargement.

*bin4-1*, *rhl2-1*, and *xs10* showed 4C/2C ratios much higher than the wild-type (2.63, 2.73, and 2.30, respectively). These mutants are known to be defective in endoreduplication [15,20]. In contrast, the *fugu2-1/fas1-5* mutant, which shows enhanced endoreduplication in leaves [19,21], showed a lower 4C/2C ratio (1.22). However, this was not consistent across all strains. Although *rpn12-1* and *cycA2;3* also showed enhanced endoreduplication [22,23], their 4C/2C ratios were 1.92 (higher than wild-type) and 1.50 (lower), respectively. In contrast, the cell size may be affected by endoreduplication in the subepidermal cells in leaves. 

**Figure 1:**
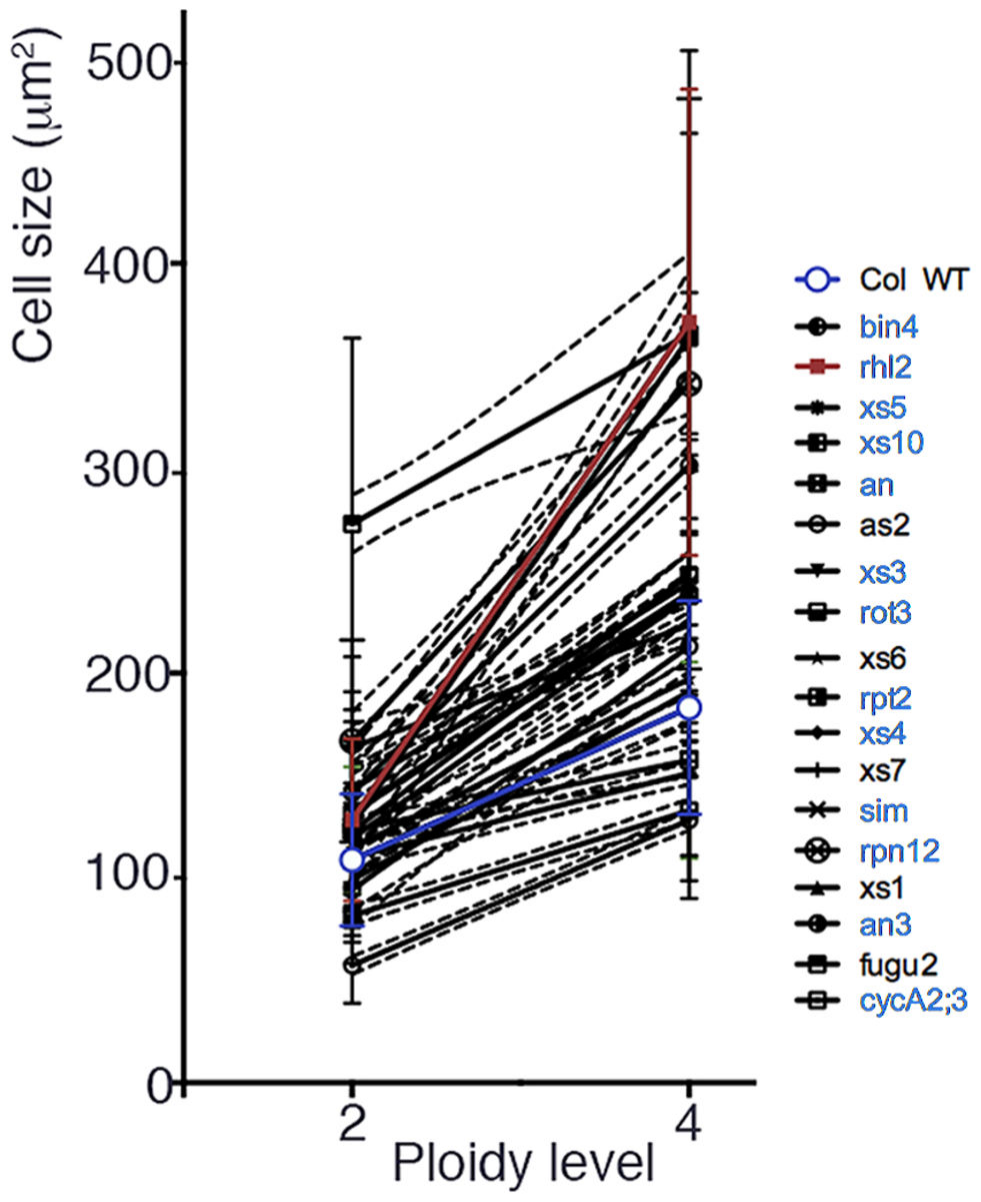
Plot of petal cell sizes in terms of projected areas in the paradermal plane against the diploid and tetraploid states. The white circle with a blue line indicates data from the wild-type (wt). The solid line indicates a regression line deduced from the data and broken lines indicate the 95% confidence intervals calculated using Prism 6. Names of mutant strains were shown in blue for which the assumption that the linear regression lines shared the same slope with that of wild type was statistically rejected (<0.01%). A regression line of the most typical case of such mutants, *rhl2*, is shown in brown, in comparison with a regression line for the wild type in blue, in the figure.

We next examined the 4C/2C ratio in another organ, petal epidermal cells, which show limited levels of endoreduplication [23,24] (Table 2). Using this approach, we eliminated the effect of endoreduplication on the observed 4C/2C ratio. As a result, the 4C/2C ratios of the petal epidermal cells were similar to leaf mesophyll cells (Table 2). To examine whether the effect of tetraploidization on cell size increase differed among strains, data sets were plotted and analyzed using Prism6 (GraphPad Software: Figure 1). As can be seen in the graph, linear regression lines for the wild-type with 95% reliability did not overlap with other strains in many cases. For example, an equation for the regression line in the *rhl2* mutant (shown in brown) was calculated as Y = 121.4X -114.5 (slope at 95% confidence, 100.4 to 142.5; Y-intercept when X = 0 at 95% confidence, -186.9 to -42.15), whereas that for the wild-type (shown in blue) was calculated as Y = 37.19X+34.37 (slope at 95% confidence, 32.78 to 41.60; Y-intercept when X = 0 at 95% confidence, 21.47 to 47.26). The assumption that these linear regression lines shared the same slope was statistically rejected (P <0.01%). Similarly, except for *xs1* (P = 15.57%), *xs6* (P = 45.67%), *xs7* (P = 5.22%), *as2* (P = 37.34%), and *fugu2/fas1* (P = 30.69%), the slopes of regression lines for all the other strains examined were judged to be significantly different from that for the wild type, respectively (P < 0.01%; Prism6, Graph Pad). 

**Table 2:** Cell size and the 4C/2C ratio of the adaxial epidermis of the petals.

Strain	Cell size (µm^2^)*		4C/2Cratio
	Diploid	Tetraploid	
Columbia wt	110.8 + 15.8	183.3 + 9.5	1.65
*brassinosteroid insensitive(bin*)*4-1* **	94.2 + 25.5	212.6 + 18.7	2.26
*root hairless (rhl*)*2-1* **	128.4 + 39.6	376.1 + 64.2	2.93
*extra small sisters (xs*)*5-1* **	112.0 + 7.6	155.2 + 16.8	1.39
*xs10-1* **	122.4 + 28.1	246.4 + 37.2	1.80
*angustifolia (an*)*-1* **	132.4 + 17.1	237.8 + 53.3	1.80
*asymmetric leaves (as)2-1*	57.2 + 9.4	128.5 + 6.0	2.25
*xs3-1* **	132.6 + 35.1	239.3 + 77.6	1.80
*rotundifolia (rot*)*3-1* **	81.8 + 8.6	133.0 + 1.7	1.63
*xs6-1*	114.5 + 29.2	198.0 + 7.6	1.73
*rpt2a-1* **	124.5 + 24.3	359.1 + 111.2	2.89
*xs4-1* **	119.1 + 27.1	237.5 + 32.2	2.00
*xs7-1*	162.8 + 3.0	223.7 + 15.8	1.37
*siamese (sim*)*-1* **	144.3 + 23.3	243.2 + 63.9	1.69
*26S proteasome regulatory particle subunit (rpn*)*12-1* **	167.0 + 15.6	333.3 + 78.2	2.00
*xs1-1*	99.0 + 27.4	190.9 + 59.9	1.93
*an3-4* **	141.8 + 4.0	294.4 + 6.0	2.00
*fugu2-1/fasciata (fas)1-5*	270.2 + 44.5	410.8 + 44.3	1.52
*cyclin (cyc*)*A2;3* **	122.5 + 12.6	157.2 + 27.3	1.28

Strains are ordered as in Table 1. Data are shown as the means + standard deviation.

^*^ At least 21 cells were measured for one petal and at least three petals were measured from each strain. Values are shown as averages.

** Mutant strain for which the assumption that the linear regression lines (shown in Figure 1) shared the same slope with that of wild type was statistically rejected (<0.01%).

### Impact of tetraploidization on the size of pollen grains

Finally the sizes of pollen grains obtained from diploid and tetraploid flowers were compared to eliminate two factors; namely, the influence of endoreduplication on the above-described phenomenon and unexpected under- or over-estimation of cell volume associated with the measured projected cell area, which does not reflect polarized cell growth in the thickness direction. Pollen grains are not subject to endoreduplication and have a spheroid cell shape; the latter allowed for precise estimation of the cell size from the projected cell area (Figure S1). The contribution of vacuole size is also limited in pollen grains. Pollen from wild-type, *rpt2a, rpn12*, and *rhl2*, all of which showed abnormal enlargement of cell size after tetraploidization in somatic cells, was evaluated. 

**Figure 2:**
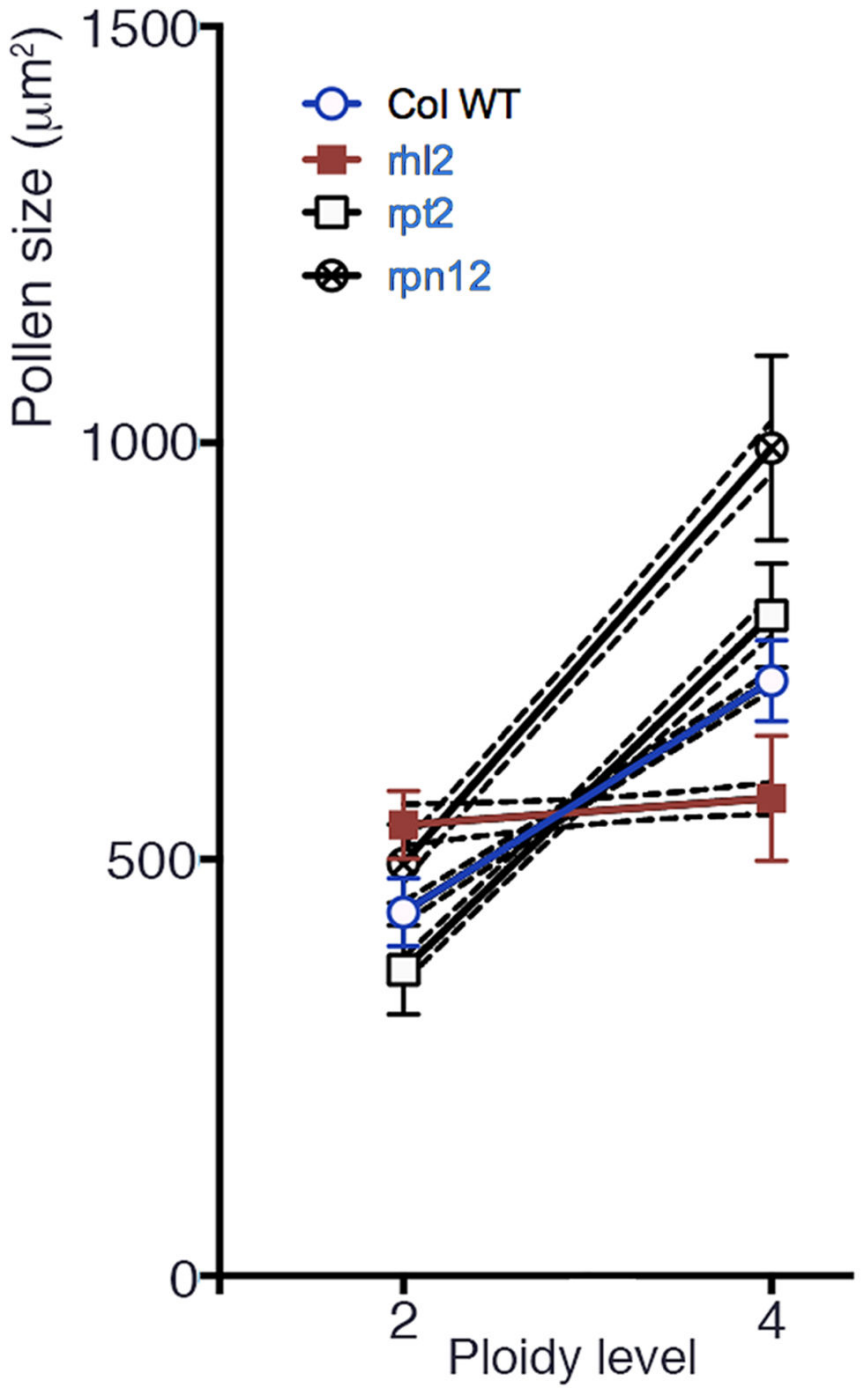
Plot of pollen grain sizes in terms of projected areas against the diploid and tetraploid states. The white circle with a blue line indicates data from the wild-type (wt). The solid line indicates a regression line deduced from the data and broken lines indicate the 95% confidence intervals calculated using Prism 6. Coloration is done as the same for the Figure 1.

The 4C/2C ratio (in terms of the ploidy of the mother plants; the ratio is 2C/1C for the pollen grains) in wild-type pollen grains was similar to 2^2/3^ = 1.587 (1.64: Table 3). In comparison, the 4C/2C ratios were much higher in *rpt2a* and *rpn12* (2.16 and 2.01, respectively), again refuting the hypothesis that ploidy-dependent cell size increase is a direct and passive result of an increase in the gene copy number. Surprisingly, the 4C/2C ratio of *rhl2* was very low (1.06), indicating that some cell-type specific control is involved in the ploidy-dependent cell size increase. The linear regression lines for the wild-type with 95% confidence (shown in blue, Figure 2) did not overlap with other strains examined (Figure 2). The assumption that these linear regression lines shared the same slope was statistically rejected (< 0.01%)

. 

**Table 3:** Pollen grain size and the 4C/2C ratio.

Strain	Cell size (µm^2^)*		4C/2Cratio
	Diploid**	Tetraploid**	
Columbia wt	436.3 + 41.0	714.3 + 48.9	1.64
*rhl2-1* ***	541.3 + 40.9	573.5 + 75.2	1.06
*rpt2a-1* ***	367.2 + 53.9	792.8 + 62.4	2.16
*26S* ** *proteasome* ** *regulatory* ** *particle* ** *subunit* (*rpn*)*12-1* *** *	494.9 + 47.3	994.0 + 110.9	2.01

* At least 20 pollen grains were measured for one strain. Data are shown as the means + standard deviation.

** Ploidy level of flowers from which pollen grains were collected. Pollen grains should read as haploid and diploid.

*** Mutant strain for which the assumption that the linear regression lines (shown in Figure 2) shared the same slope with that of wild type was statistically rejected (<0.01%).

## Discussion

The reason that increases in ploidy level increase cell volume remains unknown [8]. Galitski et al. [25] reported that when some yeast cell-cycle-related genes are proportionally down- or up-regulated, the result is altered cell size; however, later re-examination refuted this finding [9]. Thus, at this time, no changes in transcription have been attributed to ploidy-dependent cell enlargement. Instead, increased gene copy numbers may increase the amount of protein, which in turn increases cell volume. However, if all components were simply doubled, the amount of linear and two-dimensional components would be excessive [9]. On the contrary, plant cells can increase their volume without increasing the ploidy level [8,18]. Thus, cell size, cell function and ploidy level are not directly associated, and genetic regulation plays a role in ploidy level-dependent cell size. We recently found that the number of chloroplasts per cell depends on the projected cell area on the paradermal plane, but not the nuclear ploidy level [26]. For example, the *an3* mutant cells are much larger than wild-type cells without changes in endoreduplication, but contain an increased number of chloroplasts [26]. 

In the present study, I performed tetraploidization of various Arabidopsis mutants with altered cell sizes. I found that some mutants showed hyper-responses, while some were resistant to cell-volume increases after tetraploidization. This demonstrates that the cell volume is not a direct result of genome duplication, but is linked to the ploidy level through other unknown factor(s). Endoreduplication is very active in Arabidopsis leaves, but the level of endoduplication is not significantly influenced by tetraploidization [15]. Thus, the obtained data for leaf palisade cells can be interpreted without considering the effect of endoreduplication. Indeed, similar 4C/2C ratios were observed in petal epidermal cells (Table 2), in which endoreduplication is limited, and in pollen grains (Table 3), where no endoreduplicaion occurs. If polyploidy is not directly linked to cell volume (as shown here), we must reexamine the concept that endoreduplication is a prerequisite for cell enlargement in plants [27]. No endoreduplication is observed in the leaves of certain plants, such as lettuce and rice [28], but these species have various cell types with differing sizes. Moreover, changes in cell size caused by the Arabidopsis *rpt2a* mutation are not correlated with an altered level of endoreduplication among organs [23], which suggests that endoreduplication is not directly associated with cell volume. Similarly, compensated cell enlargement, which is triggered by defective cell proliferation in leaf primordia [8,17], is typically independent of the endoreduplication process [18]. Taken together, these data suggest that the ploidy level is indirectly linked to cell volume through a specific genetic pathway(s). Plant cell volume is mainly dependent on the vacuole size, which can account for more than 90% of the total cell volume. For example, it was recently shown that the *KRP2-*overxpression-dependent cell enlargement, (which is independent of the endoreduplication process) can be specifically and completely reversed by the loss-of-function mutation of *DE-ETIOLATED 3* (*DET3*), which encodes the large subunit C of the vacuolar-type H^+^-ATPase [29]. This demonstrates the importance of the ploidy-independent, vacuolar-dependent cell enlargement system. Ploidy level may affect cell volume, but the magnitude of cell expansion is likely under control of genetic pathways, as was clearly shown for leaves, petals, and pollen of the *rpt2a* and *rpn12* mutants. On the contrary, we found that *rhl2* pollen has no increase in cell volume after tetraploidization, indicative of a cell-type-specific regulation of ploidy-dependent cell volume. Further studies will increase our understanding of the mechanisms controlling plant cell size. Functional analyses of responsible genes in mutants showing altered responses of cell volume to increased ploidy level will be important to characterize this phenomenon.

## Materials and Methods

The Arabidopsis wild-type used in this study was Columbia-0. The loss-of-function *cycA2;3* mutant line (SALK_086463), *rhl2-1*, and *bin4-1* were gifts from Drs. T. Aoyama (Kyoto University) and K. Sugimoto (RIKEN). The other strains examined in this study were established previously in our laboratory under the Columbia genetic background. Plants were grown on rock wool under continuous light, as described previously [20]. Tetraploidization treatments were performed as described previously [15]. Flow-cytometry analysis was used to confirm the tetraploidy of each selected line, as described previously [30]. Some lines were further confirmed by karyotyping. For morphological and cellular analyses, first leaves dissected from 21-day-old plants were fixed in a formalin/acetic acid/alcohol solution and cleared in chloral solution [31]. Petal cell sizes were measured using a gel-printing method described previously [16]. Pollen grains were stained with lacto-phenol cotton blue, as described previously [32], to identify intact and viable grains for size measurements (Figure S1). In all measurements, the areas of images projected in the paradermal plane were measured. Microscopic measurements of leaf cell sizes were performed as described previously [33] using the program ImageJ (http://rsb.info.nih.gov/ij/; NIH, Bethesda, MD, USA). Statistical analyses were performed using Prism 6 (GraphPad Software, La Jolla, USA).

## Supporting Information

Figure S1 **Pollen grains stained with lacto-cotton blue.** Pollen grains collected from diploid (left) and tetraploid (right) flowers were stained with lacto-cotton blue. Note the simple shape of the grain. Bar, 50 μm.
(TIF)
