# Supplementary figures and images for: Does Ploidy Level Directly Control Cell Size? Counterevidence from Arabidopsis Genetics
Source: PLoS One. 2013 Dec 12;8(12):e83729. doi: 10.1371/journal.pone.0083729 (PMC3861520; doi:10.1371/journal.pone.0083729)

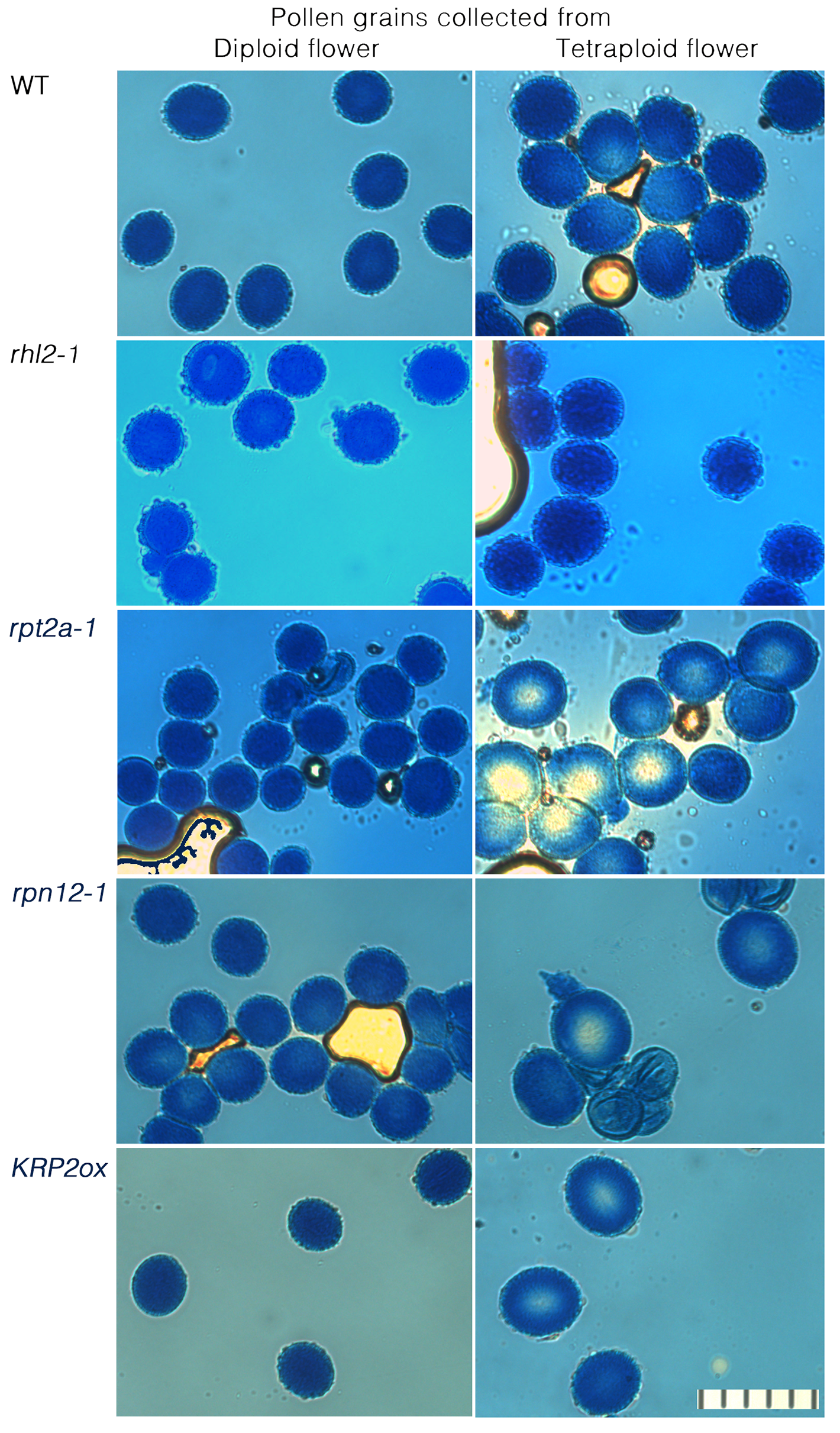

Supplement: Figure S1 — Pollen grains stained with lacto-cotton blue. Pollen grains collected from diploid (left) and tetraploid (right) flowers were stained with lacto-cotton blue. Note the simple shape of the grain. Bar, 50 μm. (TIF) [file pone.0083729.s001.tif]
